# Ectodermal Wnt6 is an early negative regulator of limb chondrogenesis in the chicken embryo

**DOI:** 10.1186/1471-213X-10-32

**Published:** 2010-03-25

**Authors:** Poongodi Geetha-Loganathan, Suresh Nimmagadda, Bodo Christ, Ruijin Huang, Martin Scaal

**Affiliations:** 1 Institute of Anatomy and Cell Biology, Department of Molecular Embryology, University of Freiburg, Albertstrasse 17, D-79104 Freiburg, Germany; 2 Present address: Department of Oral Health Sciences, Life Sciences Institute, University of British Columbia, Life Sciences Centre, 2350 Health Sciences Mall, Vancouver, BC, V6T 1Z3, Canada; 3 Present address: Insitute of Anatomy, Department of Neuroanatomy, Nussallee, D-53115 Bonn, Germany

## Abstract

**Background:**

Pattern formation of the limb skeleton is regulated by a complex interplay of signaling centers located in the ectodermal sheath and mesenchymal core of the limb anlagen, which results, in the forelimb, in the coordinate array of humerus, radius, ulna, carpals, metacarpals and digits. Much less understood is why skeletal elements form only in the central mesenchyme of the limb, whereas muscle anlagen develop in the peripheral mesenchyme ensheathing the chondrogenic center. Classical studies have suggested a role of the limb ectoderm as a negative regulator of limb chondrogenesis.

**Results:**

In this paper, we investigated the molecular nature of the inhibitory influence of the ectoderm on limb chondrogenesis in the avian embryo in vivo. We show that ectoderm ablation in the early limb bud leads to increased and ectopic expression of early chondrogenic marker genes like *Sox9 *and *Collagen II*, indicating that the limb ectoderm inhibits limb chondrogenesis at an early stage of the chondrogenic cascade. To investigate the molecular nature of the inhibitory influence of the ectoderm, we ectopically expressed *Wnt6*, which is presently the only known *Wnt *expressed throughout the avian limb ectoderm, and found that *Wnt6 *overexpression leads to reduced expression of the early chondrogenic marker genes *Sox9 *and *Collagen II*.

**Conclusion:**

Our results suggest that the inhibitory influence of the ectoderm on limb chondrogenesis acts on an early stage of chondrogenesis upsteam of *Sox9 *and *Collagen II*. We identify *Wnt6 *as a candidate mediator of ectodermal chondrogenic inhibition in vivo. We propose a model of Wnt-mediated centripetal patterning of the limb by the surface ectoderm.

## Background

The limbs of tetrapods form as mesenchymal protrusions of the somatic lateral plate mesoderm covered by an ectodermal sheath. The cartilaginous anlagen of the limb skeletal elements form from the centralmost region of the limb mesenchyme in a process called chondrogenesis (reviewed in [1]). Chondrogenesis starts by enhanced proliferation of the central limb mesenchyme, which leads to local mesenchymal condensations. One of the earliest markers of these presumptive chondrocytes is the SRY-related transcription factor *Sox9 *[2-4]. Subsequently, *Sox9 *- positive chondroprogenitor cells produce cartilage-specific extracellular matrix (ECM) components like Aggrecan and Collagen II and undergo terminal differentiation into chondrocytes. Later in development, the cartilaginous matrices of the skeletal elements become vascularized and undergo ossification.

Interaction of limb mesenchyme and ectoderm is important in the regulation of patterning, morphogenesis and differentiation of the limb bud [5,6]. The least understood patterning event is the centripetal patterning of the limb, which lays down, from the outside to the inside, epidermis, dermis, musculature, and skeleton. Into the resident limb mesenchyme, skeletal muscle presursor cells immigrate from the somites and arrange around the central chondrogenic mesenchyme to form the limb musculature in several layers [7,8]. The marginal mesenchyme in close contact with the overlying ectoderm forms the connective tissue of the dermis.

A major signaling center in this process is the ectoderm. The subectodermal mesenchyme, which will form the dermal layer of the skin, retains its non-condensed mesenchymal morphology, whereas in deeper cell layers premyogenic and prechondrogenic condensations occur. This regionalization of condensed and non-condensed mesoderm appears to be under the control of the ectoderm, since factors produced by the ectoderm prevent differentiation of nearby mesoderm to cartilage [9-11]. Classical ablation experiments have shown that in the absence of the limb ectoderm, connective tissue and cartilage develop whereas skeletal muscle differentiation is blocked [12,13], indicating a muscle-promoting activity of the limb ectoderm. In an earlier study, we provided evidence that the muscle-promoting property of the limb ectoderm is based on ectodermal Wnt signaling, namely Wnt6, which promotes Myf-5-dependent myogenesis [14]. In the absence of ectodermal Wnt signaling, the somitic precursor cells are incapable to form muscle, whereas they are still able to form endothelia [15].

In the paraxial mesoderm, Wnt6 is known to act via the canonical Wnt pathway, in which, upon Wnt ligand binding, a transmembranous Frizzled receptor triggers an intracellular signaling cascade leading to the accumulation of cytoplasmic beta-catenin and the activation of the LEF/TCF-transcription complex to promote target gene expression [16,17]. A number of studies indicate that canonical Wnt signaling suppresses chondrogenic differentiation [18-20], probably by direct interaction of beta-catenin with the early chondrogenic marker Sox9 [21,22].

To date, the molecular nature of the cartilage-inhibiting signals from the ectoderm, and the role of these signals during the molecular regulation of cartilage formation in the avian limbs, have not been established. Recently, it has been shown that Wnt3a along with FGF signaling is able to inhibit limb chondrogenesis [23]. In this study, we identified Wnt6 to be an ectodermal factor inhibiting limb chondrogenesis in the chick embryo. We found that Wnt6, which is secreted by the limb ectoderm, inhibits cartilage formation at an early stage of chondrogenesis upstream of Sox9. We propose a model that ectodermal Wnt signaling promotes myogenesis, but inhibits cartilage formation outside the centralmost limb mesenchyme, thus positioning the skeletal anlagen central to the muscular sheath of the limb. This implies a centripetal gradient of ectodermal Wnt signaling involved in limb pattern formation.

## Results

### Limb ectoderm inhibits chondrogenesis at an early stage upstream of Sox9

**Figure 1:**
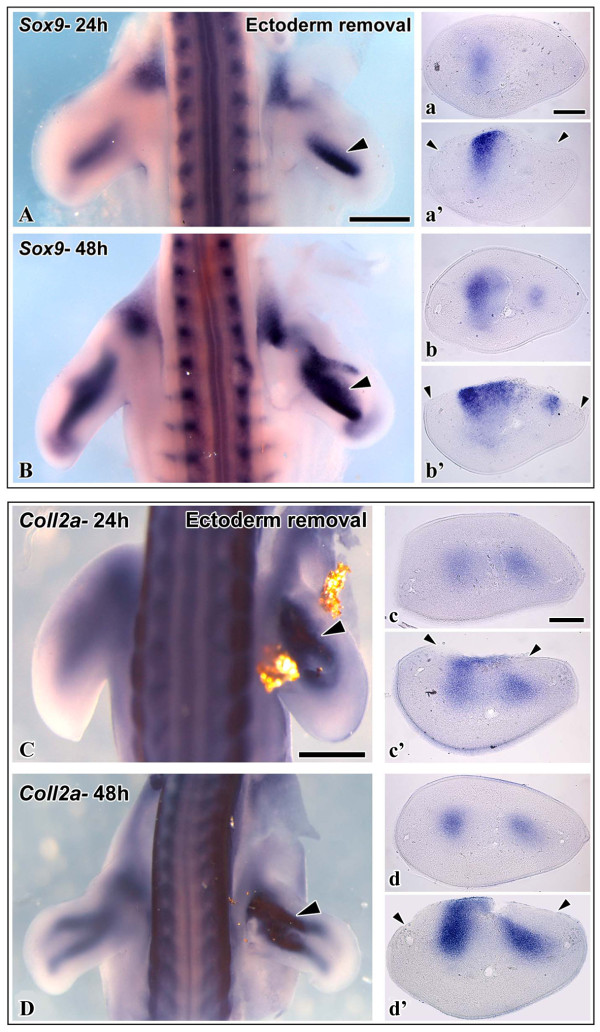
**In situ hybridizations of chicken wing buds with probes against the chondrogenic markers *Sox9 *(A, B) and *Coll2a *(C, D), after removal of the dorsal ectoderm at HH-stage 20-21 and 24 or 48 h reincubation**. On the left side is the control limb, the right limb has been manipulated. Arrowheads indicate the site of ectoderm removal. A Expression of *Sox9 *after 24 h. Expression is stronger on the operated side. a Transverse section of the control limb showing low *Sox9 *expression. a' Transverse section of the operated limb showing strong *Sox9 *expression, which is extending ectopically towards the dorsal surface of the limb. B Expression of *Sox9 *after 48 h. Expression is stronger on the operated side. b Transverse section of the control limb showing normal *Sox9 *expression. b' Transverse section of the operated limb showing enhanced *Sox9 *expression, which is extending ectopically towards the dorsal surface of the limb. C Expression of *ColIIA *after 24 h. Expression is stronger on the operated side. Dislocated fragments of gold foil are still visible. c Transverse section of the control limb showing low *ColIIA *expression. c' Transverse section of the operated limb showing strong *ColIIA *expression, which is extending ectopically towards the dorsal surface of the limb. D Expression of *ColIIA *after 48 h. Expression is stronger on the operated side. d Transverse section of the control limb showing normal *ColIIA *expression. d' Transverse section of the operated limb showing enhanced *ColIIA *expression, which is extending ectopically towards the dorsal surface of the limb. Scale bar 500 μm.

It is established from in vitro studies that the limb ectoderm is an inhibitor of chondrogenesis [9,11]. However, the molecular mechanism of the inhibitory activity of the ectoderm is still unclear. In order to investigate at what stage of the chondrogenic cascade ectodermal signals interfere with limb skeletal development in the chick, we removed the dorsal ectoderm of HH-stage 20 - 21 embryo wing buds, and analyzed the expression of a number of chondrogenic marker genes after 24 h and 48 h of reincubation. We found that ectoderm removal leads to a robust upregulation of the early chondrogenic marker genes *Sox9 *(n = 9 for 24 h, n = 7 for 48 h) and *ColIIA *(coding for Collagen II, n = 8 for 24 h, n = 9 for 48 h) (Fig. 1). Moreover, the expression domain of both markers was expanded towards the dorsal margin of the limb, where the ectoderm had been removed. This argues for an inhibitory role of the dorsal surface ectoderm during the earliest stages of chondrocyte specification.

### Ectodermal Wnt6 inhibits limb chondrogenesis upstream of Sox9

**Figure 2:**
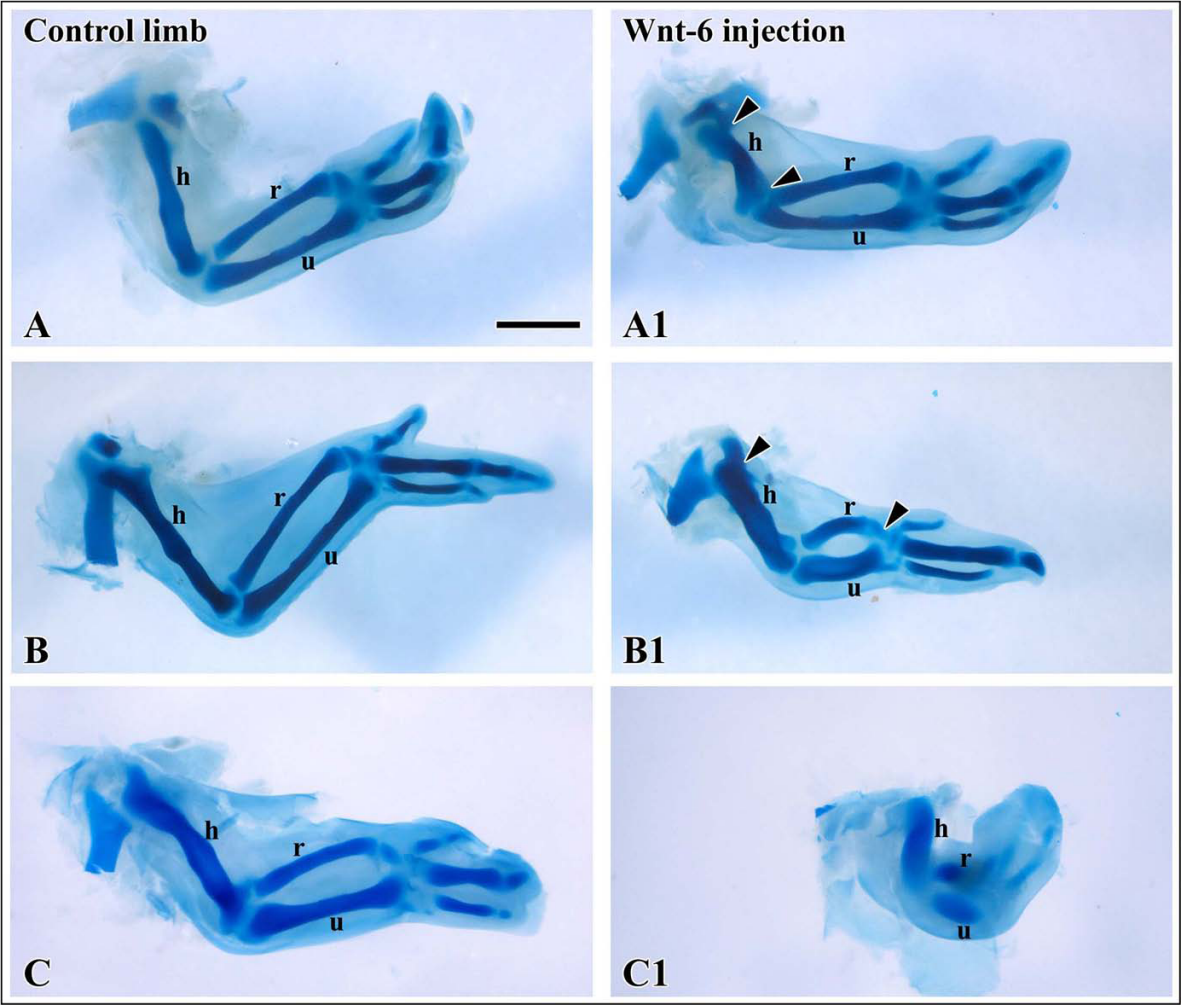
**Alcian-blue staining of the cartilaginous skeleton of embryonic chicken wings**. A-C Control wings. A1-C1 Wings after injection of Wnt6-CHO cells at HH-stage 20-21 and 4 days reincubation. Skeletal elements are shorter in length compared to the control wings. A1 to C1 illustrate increasingly severe defects depending on the quantity and spreading of injected cells, A1 being the mildest, C1 being the severest phenotype. A1 only the humerus is affected. B1 stylopod and zeugopod are affected, C1 the entire limb is affected. Scale bar 500 μm.

**Figure 3:**
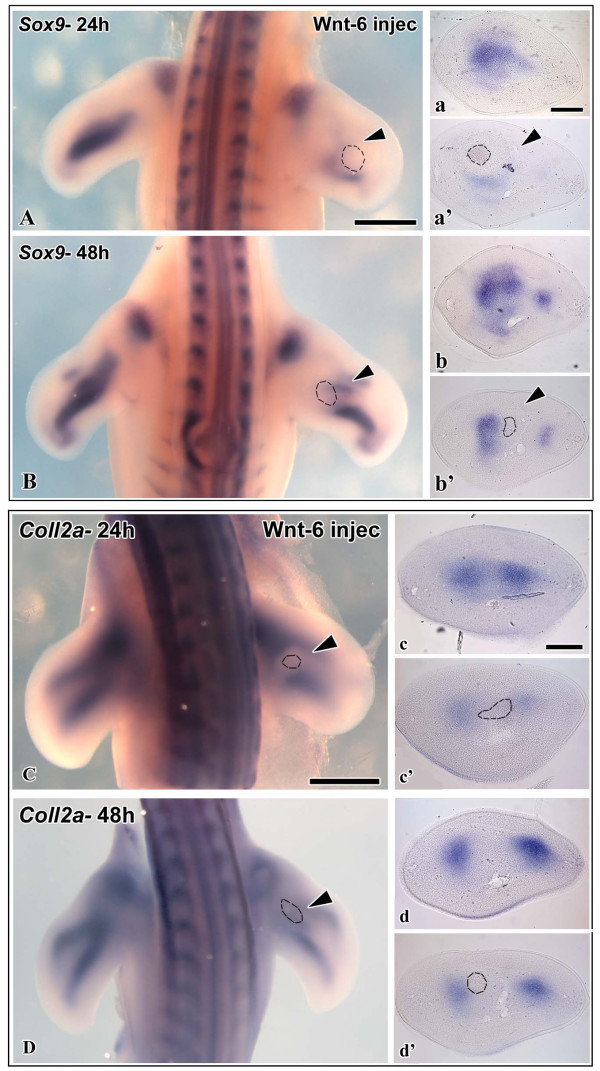
**In situ hybridization of chicken wing buds with probes against chondrogenic markers *Sox9 *(A, B) and *Coll2a *(C, D), after injection of Wnt6-CHO cells at HH-stage 20-21 and 24 or 48 h reincubation**. On the left side is the control limb, the right limb has been manipulated. Dotted line indicates the site of injection. A Expression of *Sox9 *after 24 h. Expression is severely reduced on the operated side. a Transverse section of the control limb showing normal *Sox9 *expression. a' Transverse section of the operated limb showing weak *Sox9 *expression. B Expression of *Sox9 *after 48 h. Expression is reduced on the operated side. b Transverse section of the control limb showing normal *Sox9 *expression. b' Transverse section of the operated limb showing reduced *Sox9 *expression. C Expression of *ColIIA *after 24 h. Expression is locally reduced on the operated side. c Transverse section of the control limb showing normal *ColIIA *expression. c' Transverse section of the operated limb showing weak *ColIIA *expression. D Expression of *ColIIA *after 48 h. Expression is reduced on the operated side. d Transverse section of the control limb showing normal *ColIIA *expression. d' Transverse section of the operated limb showing reduced *ColIIA *expression. Scale bar 500 μm.

**Figure 4:**
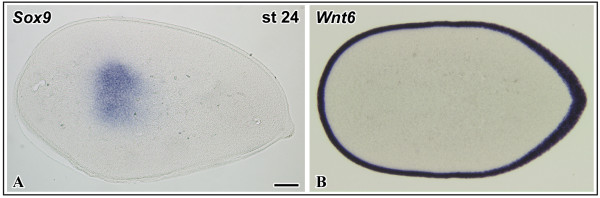
**Comparison of the expression domains of Sox9 and Wnt6 in the limb.** Slightly oblique transverse sections of wing buds at HH-stage 24. A Sox9 expression is limited to the chondrogenic region, which is located in the center of the limb bud mesenchyme. B Wnt6 is expressed in the entire circumference of the limb bud ectoderm. Scale bar 500 μm

We next searched for signals mediating the inhibitory effect of limb surface ectoderm on avian chondrogenesis. We showed earlier that Wnt6 is an ectodermal signaling molecule promoting limb myogenesis [14]. As Wnt6 is expressed throughout the limb ectoderm in chick at stages of early chondrogenesis [24,25], it is a good candidate gene to study Wnt-mediated inhibition of chondrogenesis in vivo. We injected CHO-Wnt6-transgenic cells underneath the ectoderm of wing buds at the same stages as described above for ectoderm ablation. After injection of Wnt-6 cells in HH stage 19 - 21 wing buds, Alcian blue staining after 4 days of reincubation revealed a drastic shortening of the proximal skeletal elements. The severity of the reduction in limb size depended on the amount of Wnt-producing cells applied, arguing for a dose-dependent action of Wnt signaling (Fig. 2, n = 18). Likewise, we monitored the expression of the early marker genes, *Sox9 *(n = 7 for 24 h, n = 6 for 48 h) and *ColIIA *(n = 7 for 24 h, n = 9 for 48 h), after Wnt6-cell-injection at 24 h and 48 h post surgery. Conversely to the ectoderm ablation results shown above, we observed a severe downregulation of both, *Sox9 *and *ColIIA *expression, in the chondrogenic regions of the manipulated wing buds (Fig. 3). Injection of control cells did neither change skeletal morphology, nor alter the expression of any marker gene examined (data not shown). This suggests that Wnt6 can mimick the inhibitory effect of the ectoderm on early stages of chondrogenesis. Given its expression throughout the limb ectoderm at the stages examined (Fig. 4), Wnt6, possibly together with other Wnts with yet unknown ectodermal expression, is a likely candidate to inhibit limb chondrogenesis in vivo.

In summary, our results provide evidence that chondrogenesis in the avian limb bud is negatively regulated by Wnt6-signaling from the surface ectoderm, which is acting on the earliest stages of limb chondrogenesis upstream of Sox9, thus limiting cartilage formation to the prospective skeletal anlagen in the centralmost limb mesenchyme.

## Discussion

Our results show that the limb ectoderm is a negative regulator of the earliest stages of chondrogenesis in developing chick limbs upstream of Sox9. We furthermore demonstrate that Wnt6, which is, in the chick, expressed in both dorsal and ventral limb ectoderm [24,25], inhibits chondrogenesis upstream of *Sox9 *expression, arguing for an early inhibiting activity of ectodermal Wnt6 prior to chondrocyte differentiation. At present Wnt6 is the only Wnt known to be expressed throughout the avian limb ectoderm [25] (Fig. 4). Our data do not exclude that other Wnts, like Wnt7a expressed in the dorsal ectoderm [26] or other yet unidentifed ectodermal Wnts, also participate in this function. This redundancy is very probable in the mouse, where six Wnt genes (Wnt3, Wnt4, Wnt6, Wnt7b, Wnt9b, Wnt10a) have been described to be coexpressed in the limb ectoderm [27], and several Wnts, including Wnt3a, have been shown to be inhibitors of chondrogenesis [23,28].

A regulatory role of the ectoderm during differentiation of the limb mesenchyme has already been proposed by Blechschmidt in 1963 [29]. Classical studies have experimentally confimed a role of the limb ectoderm during cartilage differentiation [30-32], whereas a regulatory role of the muscle anlagen on skeletogenesis can be excluded as muscle-less limbs form normal skeleton [33]. *In vitro *investigations have suggested that the limb ectoderm produces a diffusible factor which inhibits chondrogenic differentiation in the underlying mesenchyme. Limb ectoderm from stage 23/24 wing buds has been shown to inhibit cartilage differentiation of cultured limb mesenchyme cells even without direct contact but acting over some distance, thus arguing for a secreted mechanism of inhibition [9,10]. This is in line with the long range inhibitory activity of ectodermal Wnt6 in the limb suggested by our experiments.

The sequential steps of chondrogenesis are characterized by the expression of typical marker genes. Initially, the uniform limb mesenchyme aggregates in the central core of the limb mesenchyme to form precartilaginous mesenchymal condensations, which roughly presage the future skeletal elements. These prechondrocytic mesenchymal cells produce high levels of hyaluronic acid and cell adhesion proteins like N-CAM and N-Cadherin. During chondrogenic differentiation, the nascent chondrocytes express the nuclear transcription factor Sox9, which is required for the expression of the cartilage-specific marker gene *ColIIA1*, and start to deposit the cartilage matrix including Collagens II, IX, XI, and aggrecan. Subsequently, the balance between chondrocyte differentiation and proliferation is regulated by interaction of members of the BMP, FGF and Ihh pathways, which eventually leads to chondrocyte hypertrophy and osteogenesis (reviewed in [1]).

Akiyama et al. [34] have shown that Sox9 is required during at least two steps of cartilage formation, first during mesenchymal condensation and second, as an inducer of the related factors Sox5 and Sox6, during overt chondrocyte differentiation. In line with this, early inactivation of Sox9 leads to a loss of cartilage and bone [34]. Canonical Wnt signaling, which is known to inhibit chondrogenesis [18,19], is antagonizing Sox9 activity at posttranslational level [21]. Accordingly, forced expression of beta-catenin, a member of the canoncial Wnt pathway, in prechondrogenic cells leads to shortened or missing skeletal elements [19], a skeletal phenotype similar to the skeletal defects described here after Wnt6 overexpression. Moreover, stabilization of beta-catenin in mouse limbs leads to repression of *Sox9*, whereas deletion of beta-catenin results in an expansion of the *Sox9 *expression domain in the limb bud mesenchyme [35]. This argues for the hypothesis that the inhibitory effect of Wnt6 on chondrogenesis observed in this report could be transduced by the canoncial Wnt pathway, as has been described for the epithelializing activity of Wnt6 in the somites [17]. However, as Wnt6 has also been shown to act via non-canonical signaling [36], further studies on the downstream events leading to chondrogenic inhibition will be required to substantiate this hypothesis.

As we found that Wnt signaling not only inhibits *Sox9 *expression, but also expression of the early Sox9 target gene *ColIIA*, we argue that Wnt6 signaling negatively regulates chondrogenesis at a very early step, likely during mesenchymal condensation. This is in agreement with a recent study identifying *Sox9 *as a target of Wnt3a -mediated inhibition of cartilage formation [23].

Interestingly, also overexpression of Sox9 has been observed to result in shortened skeletal elements, likely due to inhibition of cell proliferation and differentiation [21]. This illustrates that the chondrogenesis-promoting activity of Sox9 is finely balanced. As activated beta-catenin and Sox9 are interacting in a negative feedback loop [21], correct Sox9 activity depends on an appropriate level of canonical Wnt signaling.

In accordance with this, we report here that the severity of limb skeletal defects is correlated with the amount of Wnt6 expressing cells implanted. This finding, and data from earlier work from our laboratory [14], argue for a fine-tuned, dose-dependent regulatory activity of ectodermal Wnt signaling on the development of both, muscle and cartilage. Our results support a model of centripetal patterning of the limb by ectodermal Wnt signaling: Within the peripheral limb mesenchyme, which is close to the ectodermal source of Wnt6, immigrated muscle precursor cells originating from the somites receive high doses of Wnt ligand, and differentiate into muscle [14], whereas the chondrogenic pathway is inhibited. The deeper, centrally located autochthonous mesenchyme, which is farther away from the ectodermal source of Wnt6, receives low doses of Wnt ligand, thus allowing for *Sox9 *expression and cartilage formation in the central limb. Our data are in line with a previously proposed model combining Wnt and FGF signaling in proximodistal and centripetal limb patterning [23]. In extension to this model, our results indicate that ectodermal Wnt signaling inhibits chondrogenesis upstream of Sox9, and provide in vivo data identifying Wnt6 as a candidate inhibitor. Recent results indicate that in addition to the Wnt gradient, local expression of Wnt-inhibitors like Wif-1 at the cartilage-mesenchyme-interface impedes Wnt-mediated inhibition of cartilage formation in the skeletogenic regions [37], possibly thus sharpening the circumference of the forming skeletal elements. Using Axin2^lacZ/+ ^mouse mesenchyme as reporter, Nusse and coworkers measured the reach of ectodermal Wnt signaling leading to a mesenchymal intracellular response to be 100 μm, which is in line with a Wnt-dependent centripetal patterning activity originating in the ectoderm [23]. Thus our data support the hypothesis that ectodermal Wnt signaling acts on the limb mesenchyme in a centripetal dosage gradient which is involved in specifying mesenchymal differentiation into peripheral muscle and central cartilage.

## Conclusion

In this paper, we investigated the molecular nature of the inhibitory influence of the ectoderm on limb chondrogenesis, which has been suggested by a number of classical experiments in the chick. Our data provide novel insight into two aspects of limb skeletal development:

1. So far it has been unknown at what level of the limb chondrogenic cascade the negative effect of the limb ectoderm interferes. Our results demonstrate that the ectoderm inhibits chondrogenesis at a very early stage of chondrogenesis upstream of *Sox9 *and *Collagen II*-expression.

2. Even though an inhibitory role of Wnt signaling on chondrogenesis has been known through a number of studies, no in vivo data using feasible candidate Wnts have been available. To our knowledge Wnt6 is presently the only Wnt expressed throughout the limb ectoderm at the critical stages of early chondrogenesis. We present in vivo data showing that injection of Wnt6-expressing cells into chick limb buds inhibits chondrogenesis upstream of *Sox9 *and *Collagen II*-expression.

Together, our data suggest that Wnt6 is a candidate mediator of the inhibitory influence of the ectoderm on limb chondrogenesis. We propose a model that ectodermal Wnt signaling limits chondrogenesis to the central limb mesenchyme, whereas it promotes myogenesis in the peripheral mesenchyme, thus regulating centripetal pattern formation in the avian limb bud.

## Methods

### Preparation of chick embryos

Fertilized chicken eggs were incubated at 38°C, and the embryos were staged according to Hamburger and Hamilton [38].

### Removal of limb ectoderm

For ectoderm removal, the ectoderm was stained with nile blue sulphate in ovo using a blunt glass needle coated with 2.5% agarose containing 2% nile blue. The ectoderm (of HH- stage 20-21) was peeled from the mesenchyme on the dorsal side of the forelimb bud as far as possible towards the edges without disturbing the apical ectodermal edge [14,15]. After removal of ectoderm, the limb was covered with gold foil to prevent regeneration of the ectoderm. Embryos were reincubated for 24 h/48 h, fixed and processed for whole mount *in situ *hybridization.

### Injection of Wnt-6 cells

Transfection and processing of CHO cells producing Wnt-6 protein were done as described earlier [14]. CHO control cells were used in parallel. Confluent cultures were harvested, cells were washed in phosphate-buffered saline (PBS), pelleted and resuspended in a minimal volume of medium. Experiments were performed on embryos at HH- stage 19-21. For cell injection, the ectoderm of the limb was punctured with a tungsten needle and concentrated cell suspensions were locally applied with a micropipette. The relative amount of cells injected and the localization of cells was monitored in whole mount embryos by careful inspection of injected embryos after surgery and prior to staining. Embryos were reincubated for various time periods (1-4 days) and processed for Alcian blue staining or for whole mount *in situ *hybridization.

### Whole-mount in situ hybridization

Embryos were washed in PBS and fixed overnight in 4% paraformaldehyde at 4°C, washed twice in PBT, dehydrated in methanol and stored at -20°C. Whole-mount *in situ *hybridization was performed as previously described [39]. Selected embryos were embedded in 4% agar and sectioned with a vibratome at 50 μm. The following probes were used in this study:*Sox9 *(381 bp, kindly provided by Dr. Craig Smith, Melbourne, AUS), chicken *Collagen IIA *(890 bp, kindly provided by Dr. William Upholt, Farmington, CT).

### Alcian blue cartilage staining

Whole-mount alcian blue staining was performed to visualize the skeletal phenotype of the manipulated embryos. Specimens were firstly stained with 0.015% alcian blue in 80% ethanol and 20% acetic acid for 1-3 days, fixed and dehydrated in ethanol for 1 day, and cleared and stored in 100% methylsalicylate [40].

## Authors' contributions

PG-L performed the largest part of the experiments and participated in the design of the study and in the writing of the manuscript. SN participated in the performance of the experiments and documentation of data. RH and BC participated in the design of the study, evaluation of results and interpretation of data. MS participated in the design of the study, evaluation of results and interpretation of data, and wrote the manuscript. All authors read and approved the final manuscript.
